# Sulfur fumigation of botanical drugs: impact on chemical composition and pharmacological properties, and advances in detection technologies

**DOI:** 10.3389/fphar.2025.1635850

**Published:** 2025-07-18

**Authors:** Weiyi Xu, Hongyu Jin, Ying Wang, Feng Wei, Jing Liu

**Affiliations:** Institutes for Control of Chinese Traditional Medicine and Ethnic Medicine, National Institutes for Food and Drug Control, Beijing, China

**Keywords:** sulfur fumigation, chemical metabolites, pharmacological impacts, SO2 redisues, detection technologies

## Abstract

Traditional Chinese medicine (TCM), a fundamental aspect of traditional medicine, is highly regarded for its natural efficacy and diverse applications. However, sulfur fumigation, a prevalent processing technique used to prevent insect and mold infestations, poses significant risks, including the presence of Sulfur Dioxide (SO_2_) residues, alterations in chemical compositions, diminished therapeutic effects, and heightened toxicity. As the demand for TCM continues to rise, ensuring its quality and safety becomes increasingly critical. This review explores the significance of analytical and regulatory methods for monitoring SO_2_ residues and the chemical changes induced by sulfur fumigation. The application of sulfur fumigation significantly impacts the chemical and pharmacological properties of TCM, leading to notable changes in its bioactive components. Studies show that the structure and concentration of the main components, such as ginsenosides, anthraquinones, and flavonoids are changed by this process, which decreases their pharmacological activity, which includes antibacterial, anti-inflammatory, and antioxidant properties. Additionally, the sulfur dioxide and heavy metals generated during sulfur fumigation have posed new toxicological concerns. Prolonged exposure to these residues may result in respiratory problems and potential carcinogenic effects. In recent years, researchers have created various innovative detection technologies, including fluorescent probe rapid detection methods and machine learning algorithms, to improve the precision of identifying and quantifying chemical changes in sulfur-fumigated samples. Promoting safe alternative drying methods, including natural and hot-air drying, along with desulfurization techniques, can significantly reduce the damage caused by sulfur fumigation to the chemical composition of botanical drugs. This manuscript advocates for sustainable practices to ensure the safe use of botanical drugs, protect public health, and promote responsible processing, storage, and utilization of botanical drug products. Although existing research has revealed the impact of sulfur fumigation on the chemical and pharmacological properties of certain TCM, most studies suffer from issues such as small sample sizes and lax experimental condition control, which limit the generalizability of their conclusions. Moreover, the absence of systematic research on key factors like sulfur fumigation duration and temperature makes it challenging to establish a precise quantitative relationship between the extent of sulfur fumigation and the resulting quality changes in medicinal materials.

## 1 Introduction

### 1.1 Background

Sulfur fumigation, a traditional post-harvest processing technique, has been widely utilized for the preservation of botanical drugs, especially those that serve both dietary and therapeutic purposes. This method, deeply rooted in ancient Chinese medicinal practices, was originally adopted to inhibit microbial growth, prevent insect infestations, and enhance the visual appeal of botanical drugs by preserving their color, texture, and moisture content (Zhou et al., 2019). For example, sulfur fumigation has been extensively applied to light-sensitive or moisture-sensitive botanical drugs such as *Chrysanthemum morifolium* Ramat. [Asteraceae; *Chrysanthemi morifolii* flos] (Ju Hua) and *Dioscorea opposita* Thunb. [Dioscoreaceae; *Dioscoreae oppositae* rhizoma] (DOT) (Shan Yao), effectively delaying oxidative browning and prolonging shelf life (Chan et al., 2023). Despite its practical advantages, the widespread use of sulfur fumigation has sparked considerable controversy due to its potential unintended effects on the safety and efficacy of botanical drugs (Zhou et al., 2019; Kan et al., 2011).

The combustion of sulfur generates Sulfur Dioxide (SO_2_), which not only modifies the macroscopic identification characteristics of botanical drugs but also alters their active components. Furthermore, it can contribute to chronic bronchitis, asthma, and cardiovascular diseases (Li et al., 2014; Pei et al., 2014; Sunyer, 2003; Li L. et al., 2016). Excessive residues of SO_2_ present health hazards, including respiratory irritation and potential carcinogenic effects, as noted by the World Health Organization. The fumigated botanical drugs are rich in exogenous sulfites, which can readily enter the human body through the ingestion of sulfite-containing botanical drugs or the inhalation of SO_2_ gas. Excessive sulfite levels can be detrimental to human health, indicating a significant correlation between serum sulfite levels and overall wellbeing (Maiti, 2022). Moreover, SO_2_ not only exerts toxicity on the respiratory system but can also cause oxidative damage to multiple organs (Meng, 2003).

SO_2_ interacts with bioactive components present in botanical drugs, leading to various chemical changes, including sulfonation, oxidation, and degradation. These reactions not only diminish the therapeutic properties of essential components, such as flavonoids, alkaloids, and phenolic acids, but they may also result in the formation of toxic derivatives.

As an illustration, sulfur fumigation lowers the concentration of chlorogenic acid—a critical antiviral molecule—by 30%–50% in *Lonicera japonica* Thunb. [Caprifoliaceae; *Lonicerae japonicae* flos] (LJT) (Jin Yin Hua), which is a commonly used botanical drugs for the treatment of inflammatory and infectious diseases (Liu et al., 2020). Furthermore, sulfur fumigation modifies the chemical composition of botanical drugs. For instance, in the case of *Paeonia lactiflora* Pall. [Ranunculaceae; *Paeoniae lactiflorae* radix alba] (PLP) (Bai Shao), this process can convert its bioactive component, paeoniflorin, into a sulfur-containing derivative referred to as paeoniflorin sulfite. This transformation may subsequently impact the quality, bioactivities, pharmacokinetics, and toxicities of PRA (Wang J. et al., 2024). These dual risks—reduced efficacy and increased toxicity—underscore the urgent need to reevaluate the use of sulfur fumigation in botanical drugs.

The China National Medical Products Administration, along with the U.S. Food and Drug Administration and similar agencies in South Korea and Europe, has implemented practical regulations and established standard limits for SO_2_ residues (NLM, 2020). (United States Pharmacopoeia 47, 2024; European Pharmacopoeia 11.0, 2024). These measures are designed to ensure the efficacy and safety of food and medical products that have undergone sulfur fumigation.

The fumigation of traditional Chinese medicine (TCM) with sulfur results in an increase in SO_2_ residues that is proportional to the duration of the fumigation process. However, these residues tend to decrease during the storage of samples (Liu et al., 2022). Consequently, the levels of SO_2_ residues alone do not provide an accurate measure of the extent of fumigation or the associated chemical transformations. Research indicates that there is no direct correlation between residue levels and the chemical changes induced by sulfur fumigation (Zhou et al., 2019; Kang et al., 2017; Xing et al., 2018). This suggests that significant chemical alterations can occur even in the presence of low residue levels. To comprehensively evaluate the effects of sulfur fumigation on botanical drugs, it is crucial to consider not only the SO_2_ residue levels but also their implications for botanical drug quality, efficacy, and toxicity. Unfortunately, existing standards that focus solely on monitoring residue levels do not adequately address the chemical changes induced by SO_2_ and their subsequent effects on efficacy and toxicity.

Consequently, there is a pressing need for innovative technologies and methodologies to thoroughly assess the quality of botanical drugs treated with sulfur fumigation. These advancements should be capable of identifying chemical alterations resulting from sulfur fumigation and evaluating their impact on both efficacy and safety.

This review discusses the impact of sulfur fumigation on the chemical composition and pharmacological properties of botanical drugs, highlighting how this practice can lead to significant changes in bioactive components and potential increases in toxicity. Additionally, the review examines advances in detection technologies and alternative processing methods, emphasizing the need for innovative approaches to ensure the quality and safety of sulfur-fumigated botanical drugs. Through these efforts, the review contributes to promoting responsible practices in the processing and use of traditional Chinese medicine. Nevertheless, while existing research has unveiled the impact of sulfur fumigation on certain chemical and pharmacological aspects of traditional Chinese medicinal materials, most studies are marred by issues such as limited sample sizes and lax experimental condition management, which undermines the general applicability of their conclusions. Moreover, the absence of in - depth research on key parameters like sulfur fumigation duration and temperature makes it challenging to define a precise quantitative link between the extent of sulfur fumigation and the resulting quality variations in medicinal materials.

## 2 Impact of sulfur fumigation on botanical drugs

### 2.1 Chemical alterations

In the process of fumigation, TCM are composed of complex molecules which interact with SO_2_ or sulfurous acid. Phenylpropanoids, terpenoids, saponins, and polysaccharides may also undergo several transformations. The chemical reactions lead to a reduction in the concentration of certain components, which can significantly affect the quality of the medicinal materials. Furthermore, this procedure may give rise to the synthesis of new chemicals.

**FIGURE 1:**
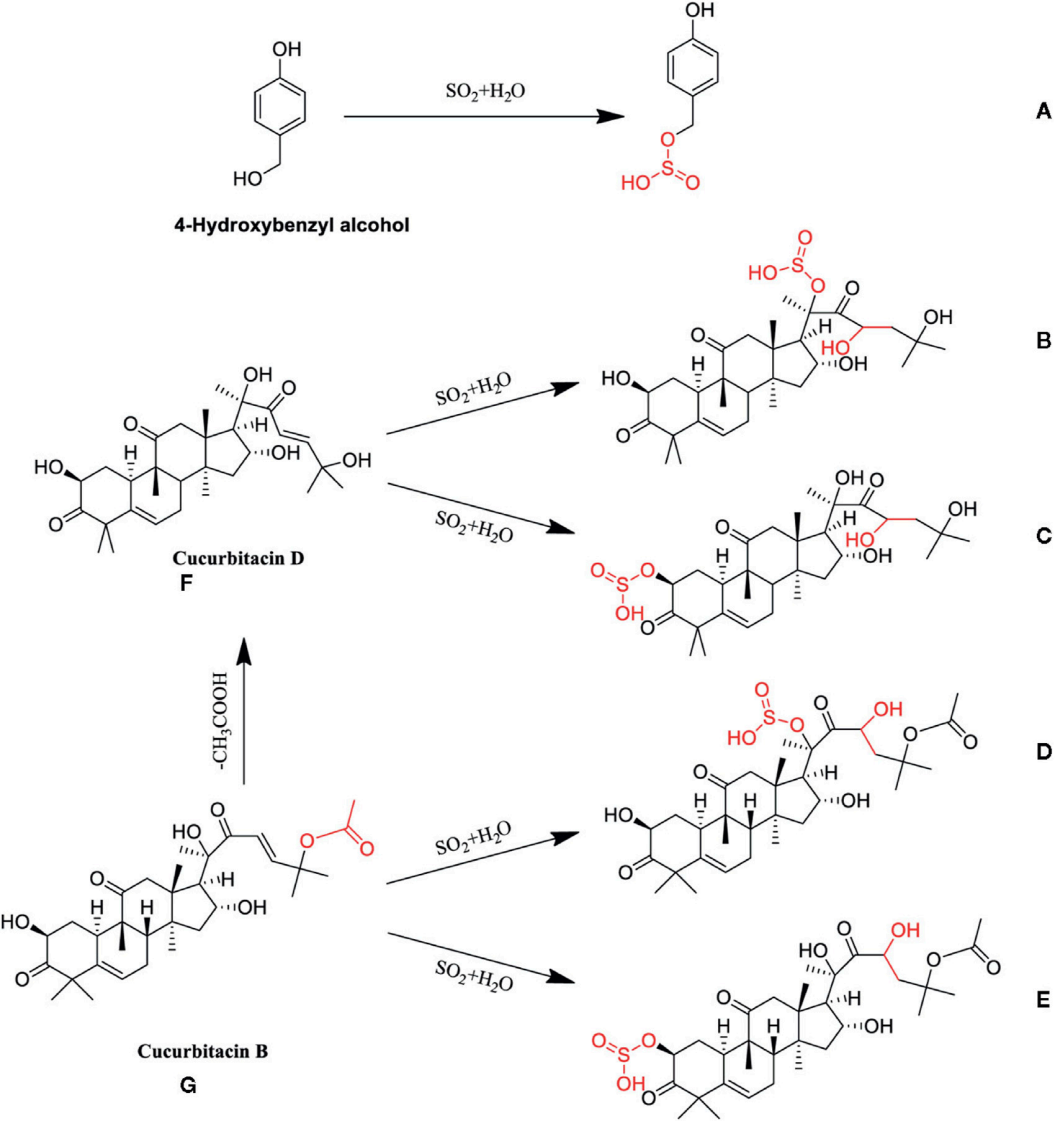
Possible chemical structure changes of the markers in TKM during sulfur fumigation. **(A)** p-Hydroxybenzyl hydrogen sulfite; **(B)** Cucurbitacin D sulfite I; **(C)** Cucurbitacin D sulfite II; **(D)** Cucurbitacin B sulfite I; **(E)** Cucurbitacin B sulfite II; **(F)** Cucurbitacin D; **(G)** Cucurbitacin B.

Sulfur fumigation exerts a significant influence on the composition of terpenoid components present in TCM. For instance, in *Atractylodes macrocephala* Kitag. [Asteraceae; *Atractylodis macrocephalae* rhizoma] (AMK) (Bai Zhu), the concentration of six terpenoid componentss markedly decreases following sulfur fumigation. Concurrently, fifteen novel terpenoid metabolites emerge, including eight sesquiterpene lactones that either incorporate sulfur or undergo dehydration condensation, such as Atractylenolide III sulfate, Isoasterolide A sulfite, and 6-hydroxy atractylenolide I sulfate (Ge et al., 2023). Similarly, the chemical profile of *Trichosanthes kirilowii* Maxim. [Cucurbitaceae; *Trichosanthis kirilowii* radix](TKM) (Gua Lou Gen) is significantly altered by sulfur fumigation. In only 1 h following treatment, fourteen different types of cucurbitacin components, including cucurbitacin B and D, diminishes, whereas sulfur-containing derivatives are produced, such as cucurbitacin D sulfite I, cucurbitacin D sulfite II, cucurbitacin B sulfite I, and cucurbitacin B sulfite II. Figure 1 shows the possible chemical structure changes of the markers in TKM during sulfur fumigation (Kang et al., 2020). Furthermore, sulfur fumigation of LJT (Jin Yin Hua) initiates sulfation and sulfonation reactions, leading to the formation of various new sulfur-containing metabolites. For example, secologanin, a compound featuring a cyclopentane moiety, undergoes sulfonation to yield its sulfonate derivative (Liu et al., 2020). Following the treatment of PLP (Bai Shao) with sulfur fumigation, multiple sulfur-containing derivatives are produced, primarily including paeoniflorin sulfonate and its isomers, as well as oxypaeoniflorin sulfonate. Additionally, this process results in the generation of endogenous metabolites such as p-cresol glucuronide (Wu et al., 2012; Kong et al., 2018). The chemical composition of *Moutan Cortex* Andr. [Ranunculaceae; *Moutan cortex*](MCA) (Mu Dan Pi) is also significantly modified by sulfur fumigation, resulting in the formation of ten new sulfonate derivatives. These include oxy paeoniflorin sulfonate, suffruticoside E sulfonate, paeoniflorin sulfonate, suffruticoside D sulfonate, phenolic acid oxy paeoniflorin sulfonate, phenolic acid paeoniflorin sulfonate, suffruticoside H sulfonate, benzoyloxy paeoniflorin sulfonate, suffruticoside C sulfonate, and benzoylpaeoniflorin sulfonate (Li X.-Y. et al., 2016). During the sulfur fumigation of *Codonopsis pilosula* Franch. [Campanulaceae; *Codonopis pilosulae* radix] (CPF) (Dang Shen), the content of atractylenolide sharply declines within the first hour of treatment (Xu et al., 2020). Sulfonation and sulfation are the main changes in the terpenoid compound transformation processes. During sulfur fumigation, terpenoid compounds can form sulfates or sulfonates either directly or indirectly. Additionally, some terpenoid compounds may initially undergo dehydration to form dehydrated compounds, which subsequently react with SO_3_ or SO_2_ to generate the corresponding sulfates or sulfonates.

**FIGURE 2:**
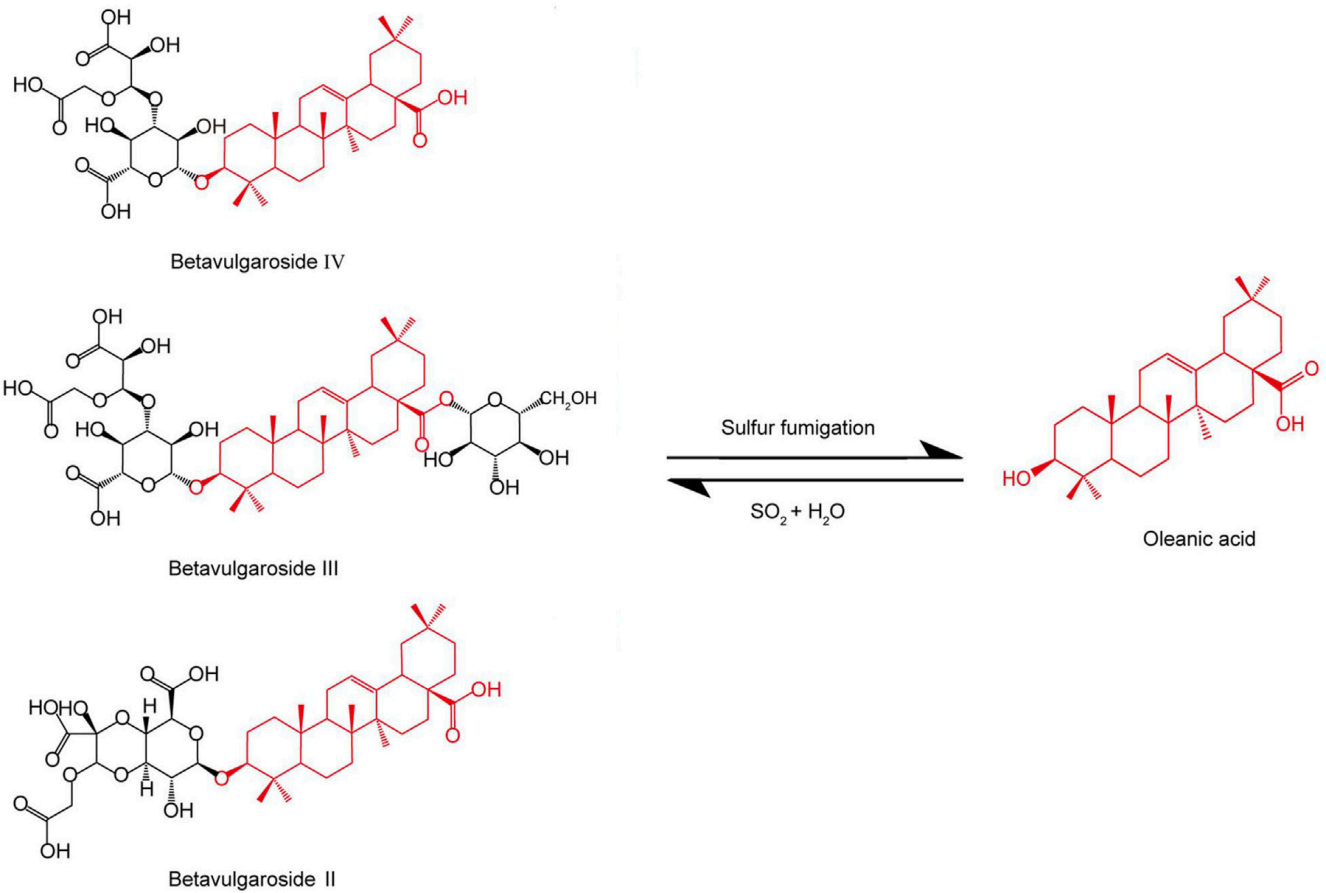
Possible chemical structure changes of the markers in ABB during sulfur fumigation.

The amount of phenylpropanoid components might change dramatically with the application of sulfur fumigation of TCM. During sulfur fumigation of *Gastrodia elata* Bl. [Orchidaceae; *Gastrodiae elatae* rhizoma](GEB) (Tian Ma), eight phenylpropanoid components—Adenosine, Gastrodin, p-Hydroxybenzyl alcohol, p-Hydroxybenzaldehyde, Parishin A, Parishin B, Parishin C, and Parishin E—show a decline in the early stages (1–2 h) of fumigation, but then gradually rebound and stabilize after 2 h (Kang et al., 2017). In *Angelica sinensis* (Oliv.) Diels [Apiaceae; *Angelicae sinensis* radix] (Dang Gui), sulfur fumigation leads to a reduction of over 50% in the content of Ferulic acid, (E)-ligustilide, Senkyunolide I, and Senkyunolide H (Duan et al., 2016). Sulfur fumigation of *Angelica dahurica* (Fisch. ex Hoffmanns. and Link) Benth. and Hook. f. [Apiaceae; *Angelicae dahuricae* radix] (ADBH) (Bai Zhi) causes a marked decrease in specific coumarin components, such as Imperatorin and Oxypeucedanin, to the point of being nearly undetectable (Wang C. et al., 2024). For *Rheum palmatum* L. [Polygonaceae; *Rhei* radix et rhizoma] (RPL) (Da Huang), sulfur fumigation results in a reduction of coumarins and benzopyranone compounds (Yan et al., 2016). Notably, sulfur fumigation of *Tussilago farfara* L. [Asteraceae; *Farfarae* flos] (TFL) (Kuan Dong Hua) generates a large number of sulfated phenylpropanoid metabolites (Wang et al., 2021). Sulfur fumigation has the potential to modify the saponin components found in TCM. In the case of *panax ginseng* C.A.Mey. [Araliaceae; *Ginseng* radix et rhizoma] (PGC) (Ren Shen) and *Panax quinquefolius* L. [Araliaceae; *Panacis quinquefolii* radix](PQL) (Xi Yang Shen), sulfur fumigation results in a reduction of original ginsenosides, such as Re, Rg1, and Rb1, while simultaneously generating numerous sulfated derivatives, including Re sulfate. It is notable that there was no linear relationship between the concentration of SO_2_ and the decrease in original ginsenosides (Zhou et al., 2019; Zhang et al., 2018; Jin et al., 2012). Additionally, following sulfur fumigation, the levels of two triterpene saponins, astragaloside III and IV, in *Astragalus membranaceus* Bunge [Fabaceae; *Astragali* radix] (AMB) (Huang Qi) also decline (Xing et al., 2018). In *Achyranthes bidentata* Blume [Amaranthaceae; *Achyranthis bidentatae* radix] (ABB) (Niu Xi), sulfur fumigation leads to a reduction in several triterpene saponins, including Betavulgaroside IV, III, and II. Figure 2 shows the possible chemical structure changes of the markers in ABB during sulfur fumigation. (Kang et al., 2018; Dong et al., 2023). Furthermore, in *Ophiopogon japonicus* Ker Gawl. [Asparagaceae; *Ophiopogonis* radix] (Mai Dong), sulfur fumigation results in the removal of certain glycosyl groups from steroidal saponins, giving rise to sulfur-containing derivatives. A total of ninety-five such derivatives, encompassing sulfates and sulfonates, have been identified (Li et al., 2024). The formation of sulfur-containing derivatives (SCDs) occurs through three primary mechanisms. First, the hydroxyl groups (-OH) of saponins react with sulfurous acid (H_2_SO_3_) or sulfuric acid (H_2_SO_4_) produced during the fumigation process, resulting in the formation of esters. Second, internal double bonds (C=C) within these compounds undergo sulfonation with sulfur trioxide (SO_3_), which involves double bond cleavage, ring addition, and the creation of unstable sulfonate intermediates that ultimately convert into vinyl sulfonates. Lastly, double bonds may also participate in radical addition reactions with H_2_SO_3_, leading to the generation of sulfonated products. Collectively, these reactions contribute to the formation of SCDs (He et al., 2023).

The polysaccharides contained in TCM can be seriously affected by sulfur fumigation. For CPF (Dang Shen), it changes both the monosaccharide composition and molecular weight distribution of polysaccharides, changing the ratios of mannose, galacturonic acid, rhamnose, glucose, galactose, and arabinose (Xu et al., 2020). In AMK (Bai Zhu), while the molecular weight distribution of polysaccharides remains unchanged, their content decreases, alongside an increase in oligosaccharides and free fructose. These changes intensify with higher SO_2_ residue levels (Ge et al., 2023). In DOT (Shan Yao), sulfur fumigation reduces the extraction rate of polysaccharides, alters their composition and structure, increases uronic acid content, and decreases total sugar content. Significant changes also occur in monosaccharide composition and glycosidic bond types (Chan et al., 2023). For PGC(Ren Shen) sulfur fumigation broadens the molecular weight distribution of polysaccharides, partially degrading them into oligosaccharides and monosaccharides. However, after 2 weeks of storage, the polysaccharide content recovers somewhat, while oligosaccharide levels decrease (Zhou et al., 2019). Si Jun Zi Tang is a traditional Chinese herbal formula, with PGC(Ren Shen) as the primary botanical drug, commonly used to treat various conditions, including colorectal cancer. Its main components are PGC(Ren Shen), AMK(Bai Zhu), *Poria cocos* (Schumach. and Thonn.) Tul. [Polyporaceae; *Poriae* fructificatio](Fu Ling), *Glycyrrhiza uralensis* Fisch. ex DC. [Fabaceae; *Glycyrrhizae* radix et rhizoma] (Gan Cao), sulfur fumigation reduces the total polysaccharide content and molecular weight in Si Jun Zi Tang, alters monosaccharide compositions, and increases oligosaccharide and free monosaccharide levels (Liu et al., 2022). These changes are likely due to the acidic conditions created by sulfur fumigation, which accelerates the degradation of polysaccharides into oligosaccharides or monosaccharides.

**FIGURE 3:**
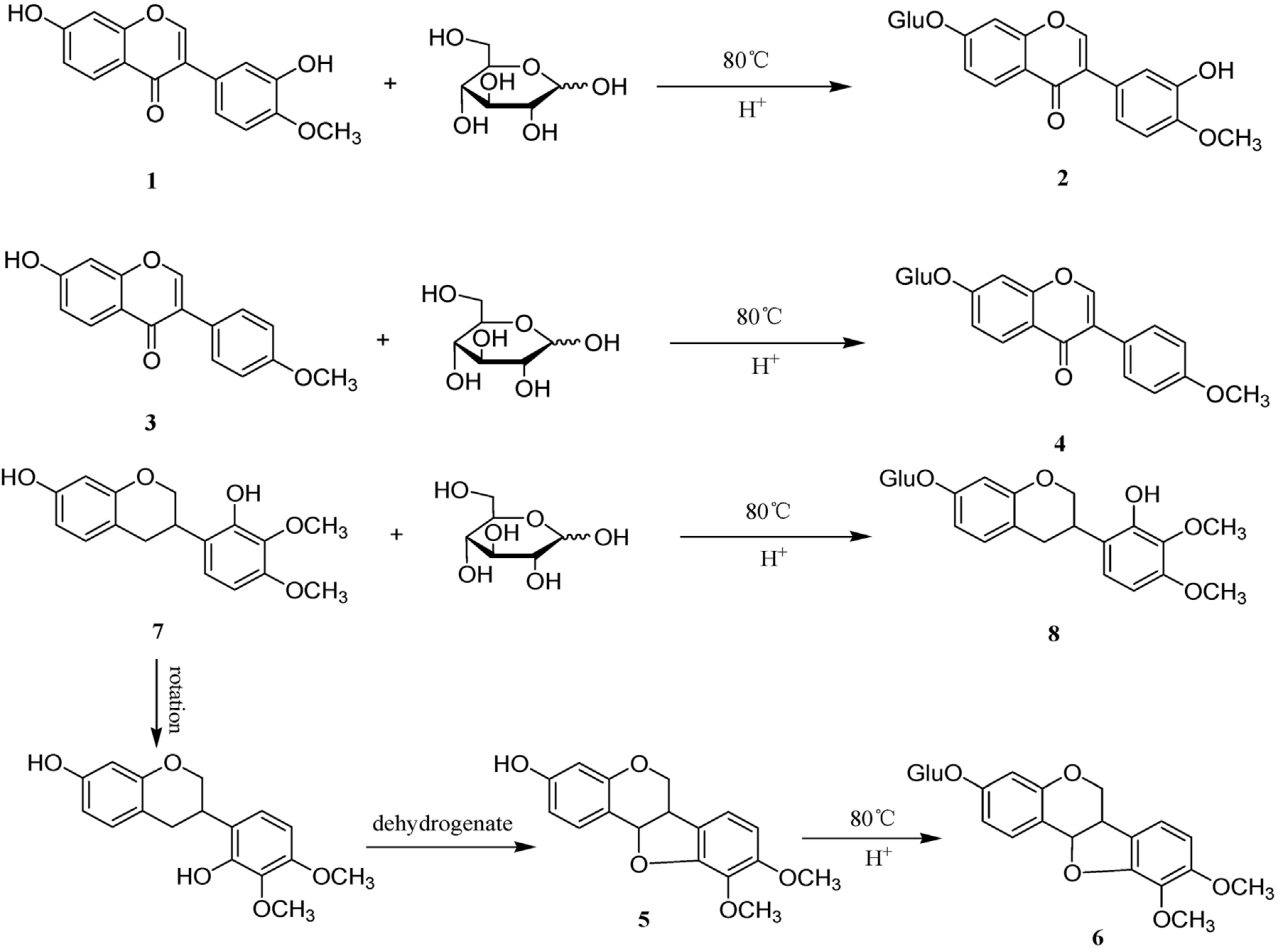
Possible chemical structure changes of the markers in AMB during sulfur fumigation. (1) Calycosin; (2) calycosin-7-glucoside; (3) formononetin; (4) ononin; (5) Methylnissolin; (6) astraisoflavan-7-O-β-D-glucoside; (7) 7,2′-dihydroxy-3′,4′-dimethoxyisoflavane; (8) 7,2′-dihydroxy-3′,4′-dimethoxy isoflavan-7-O-β-D-glucopyranoside.

Sulfur fumigation can also lead to changes in the content of flavonoid and alkaloid components in TCM. For example, after sulfur fumigation, the content of flavonoids and alkaloids in CPF(Dang Shen), such as lobetyol, baicalin, and rutin, decreases sharply (Xu et al., 2020). Sulfur fumigation can reduce the content of adenosine, an alkaloid compound in GEB(Tian Ma) (Kang et al., 2017). After sulfur fumigation, the content of two flavonoid compounds (calycosin and formononetin) in AMB (Huang Qi) decreases significantly, while the content of the corresponding flavonoid glycosides increases markedly. This indicates that flavonoid compounds undergo chemical transformations during the sulfur fumigation process. Figure 3 shows the possible chemical structure changes of markers in AMB during sulfur fumigation (Xing et al., 2018).

**FIGURE 4:**
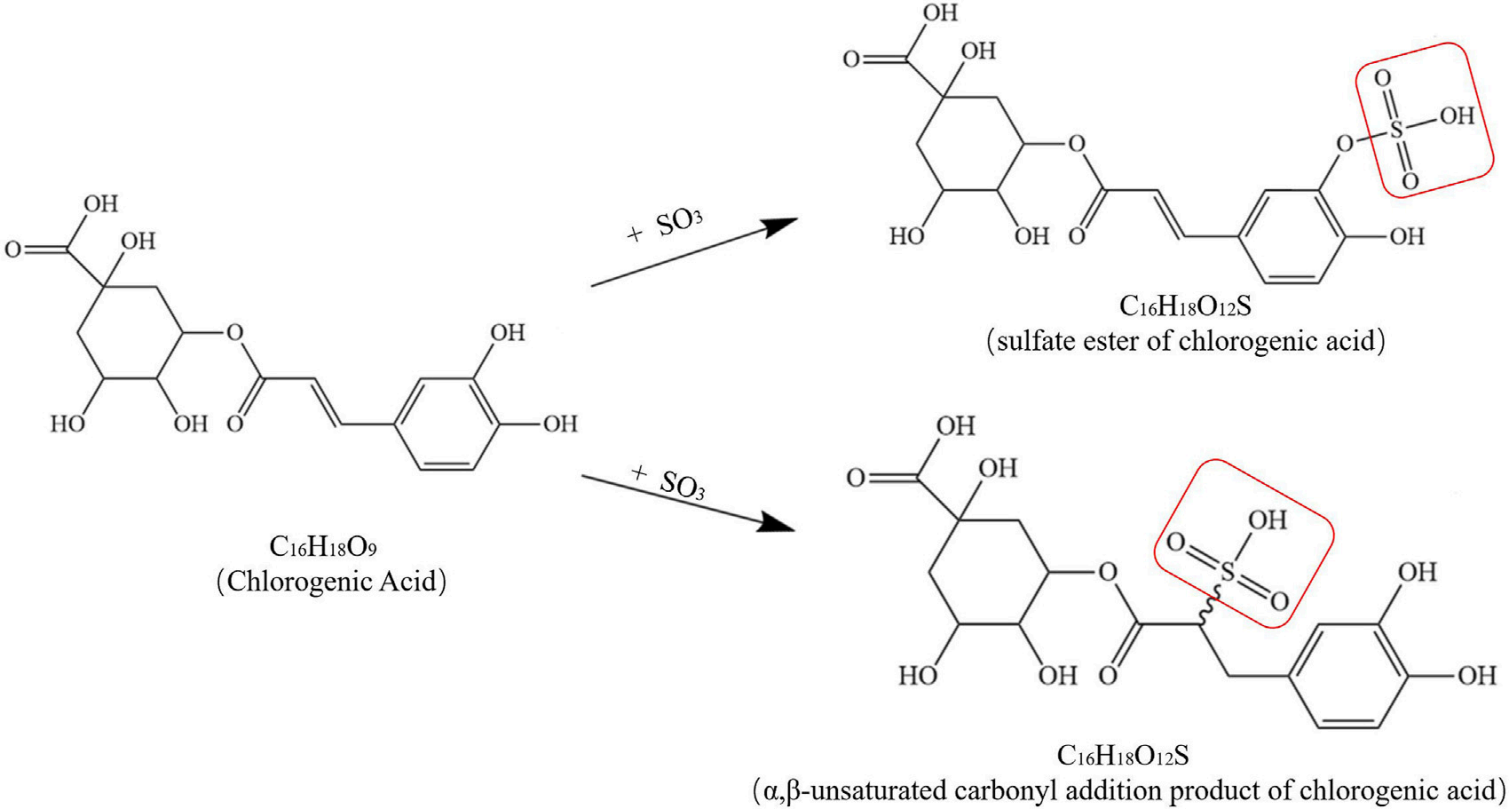
Possible chemical structure changes of 5-CQA during sulfur fumigation.

In addition, sulfur fumigation has a considerable effect on other chemical components in TCM. For instance, after sulfur fumigation, the contents of organic acids and volatile oils in LJT (Jin Yin Hua) generally decrease. Chlorogenic acid (5-CQA) is converted into sulfated products (e.g., 5-CQA sulfate), and the contents of furan, alkali, acid, aldehyde, ketone, alcohol, terpene, and ester compounds in volatile oils also decline, with some alkali and acid compounds even completely disappearing. Figure 4 shows possible chemical structure changes of 5-CQA during sulfur fumigation (Liu et al., 2020; Cai et al., 2013). In the case of RPL (Da Huang), the contents of quinone compounds (especially anthraquinone glycosides) and tannins significantly decrease or even vanish after sulfur fumigation. In particular, anthraquinone glycosides (e.g., emodin-8-O-β-D-glucoside) nearly vanish, other anthraquinone compounds decrease by between 52% and 87%, tannins (e.g., gallic acid, catechin, and cinnamoyl-O-galloyl glucose) show reductions of 67%, 71%, and 90% respectively, many tannins completely disappear after sulfur fumigation (Yan et al., 2016). Additionally, sulfur fumigation significantly alters the chemical composition of *Zingiber officinale* Rosc. [Zingiberaceae; *Zingiberis rhizoma* recens](ZOR) (Sheng Jiang), especially reducing the content of 6-gingerol and generating new metabolites such as 6-ginger sulfonic acid (Wu et al., 2018). DOT (Shan Yao) also experiences significant changes in the profile of small-molecule metabolites after sulfur fumigation. Some metabolites decrease or disappear, such as phenylalanine, tryptophan, D-pantothenic acid, 9,10,13-trihydroxy-11-octadecenoic acid, and 9,10,11-trihydroxy-12-octadecenoic acid, while the contents of other metabolites increase or new metabolites are detected, such as LysoPE (0:0/18:2), LysoPE (18:2/0:0), LysoPC (0:0/18:2), and methyl citrate. Tryptophan sulfonate is also newly detected in sulfur-fumigated samples (Chan et al., 2023).

Notably, most current studies rely on a single chemical analysis method, which fails to fully reflect the subtle changes and complex metabolic pathways of the chemical metabolites in TCM. Meanwhile, the control of key variables, such as sulfur fumigation concentration and duration, is not precise enough in experiments, which may lead to deviations in the results. Table 1 summarizes the key reaction types, mechanisms, and their relationship with functional group structures that may occur during the sulfur fumigation process, breaking down each reaction type and the functional group changes that accompany them. This allows for a more precise understanding of the specific alterations that sulfur fumigation can cause in the chemical composition of traditional Chinese medicinal materials. However, the reaction mechanisms may still be unclear and require further research to fully elucidate the reaction mechanisms.

**TABLE 1:** Core reaction types and their correlations with functional group structures.

Core reaction type	Reaction mechanism	Functional group changes	Example	References
Sulfonation	Involves the reaction of hydroxyl groups (-OH) with sulfuric acid (H_2_SO_4_) or sulfurous acid (H_2_SO_3_) to form sulfonate esters (-OSO_3_H)	-OH → -OSO_3_H	Atractylenolide III + H_2_SO_4_ → Atractylenolide III sulfate	Ge et al. (2023)
Sulfonization with SO_2_	Involves the reaction of hydroxyl groups (-OH) with sulfur dioxide (SO_2_) to form sulfonate esters (-OSO_2_H)	-OH → -OSO_2_H	Gastrodin + SO_2_ → Gastrodin sulfonate	Kang et al. (2017)
Sulfation	Involves the reaction of hydroxyl groups (-OH) with sulfuric acid (H_2_SO_4_) to form sulfate esters (-OSO_3_H)	-OH → -OSO_3_H	Genistein + H_2_SO_4_ → Genistein sulfate	Xing et al. (2018)
Oxidation	Involves the reaction of double bonds (C=C) with oxygen to form carbonyl (C=O) or carboxyl (-COOH) groups	C=C → C=O or -COOH	Ginsenoside Re + O_2_ → Oxidized ginsenoside Re	Zhou et al. (2019)
Degradation	Involves the cleavage of glycosidic bonds in polysaccharides to form low-molecular-weight products	Glycosidic bond cleavage	Dioscorea polysaccharides → Oligosaccharides + Monosaccharides	Chan et al. (2023)
Cycloaddition	Involves the reaction of double bonds (C=C) with SO_3_ to form unstable sulfonate intermediates, which ultimately convert into vinyl sulfonates	C=C → Cyclic structure + -OSO_3_H	Certain terpenoid compounds + SO_3_ → Vinyl sulfonates	Kang et al. (2020)

### 2.2 Pharmacological impacts

Sulfur fumigation of TCM can alter its chemical composition, disrupt normal pharmacokinetic processes, reduce efficacy, and pose potential toxicological risks. It may also lead to metabolic and pharmacokinetic changes, such as altered bioavailability and metabolic pathways of active metabolites.

PLP (Bai Shao) is renowned for its ability to soothe the liver and alleviate pain, making it effective in treating abdominal pain, limb pain, and headaches (NLM, 2020). However, studies indicate that sulfur fumigation can significantly diminish the analgesic activity of it. In the mouse hot-plate test, non-sulfured PLP demonstrated a significant analgesic effect at a high dose (45.4 g/kg). In contrast, sulfured PLP failed to exhibit a significant analgesic effect, even at both high (45.4 g/kg) and low doses (11.4 g/kg). This suggests that sulfur fumigation may alter the chemical composition and metabolites of PLP, thereby affecting its absorption, distribution, metabolism, and excretion in the body, ultimately weakening its analgesic effect. Specifically, sulfur fumigation significantly reduces the systemic exposure of paeoniflorin and oxypaeoniflorin, the primary analgesic compounds of PLP. The AUC (0−t) of paeoniflorin decreased from 5,575.85 ± 690.30 ng/mLh to 3,131.87 ± 642.63 ng/mLh, while that of oxypaeoniflorin decreased from 218.31 ± 37.51 ng/mLh to 134.81 ± 43.52 ng/mLh. This reduction in exposure directly undermines the analgesic effect. Moreover, sulfur fumigation leads to the formation of new sulfur-containing derivatives in PLP, such as paeoniflorin sulfonate, which has a high AUC (0−t) of 7,077.06 ± 2,232.97 ng/mLh and a Cmax of 1,641.42 ± 634.79 ng/mL. This may further interfere with the original pharmacological actions of PLP. Furthermore, in rats treated with sulfured PLP, the characteristic metabolite p-cresol glucuronide was detected in plasma, bile, urine, and feces. This indicates that sulfur fumigation may alter the homeostasis of gut microbiota and bile acids, thereby affecting drug metabolism. Such metabolic changes may be another important factor contributing to the weakened analgesic effect of sulfured PLP (Kong et al., 2018).

PGC (Ren Shen) is renowned for its various health benefits, including anti-inflammatory, anti-shock, anti-anxiety, anti-fatigue, and immune-modulating properties (Ratan et al., 2021). Research indicates that non-sulfured PGC can effectively alleviate symptoms in mice with cyclophosphamide-induced immunosuppression. This includes improvements such as an increased spleen weight index, elevated serum levels of IL-2 and IFN-γ, and a reduction in white blood cell count. In contrast, sulfured PGC does not exhibit these beneficial effects, suggesting that sulfur fumigation may compromise its ability to regulate immune function (Ma et al., 2017). Furthermore, non-sulfured PGC exhibits enhanced anti-inflammatory, anti-shock, and anti-stress properties when compared to sulfured PGC. However, these advantages tend to decrease as the storage duration of sulfured PGC increases (Zhou et al., 2019). Pharmacokinetic studies indicate that sulfur fumigation significantly diminishes the levels of ginsenosides, which are the active compounds in PGC, resulting in markedly reduced systemic exposure within the body. Following a single dose, five ginsenosides (Rb1, Rb2, Rc, Rd, and Rg1) along with two metabolites were identified in the plasma of rats after the administration of non-sulfured PGC; however, none were detected following the administration of sulfured PGC. For multiple dose, in the sulfured PGC, only two ginsenosides, Rb1 and Rb2, were detected, and at significantly lower concentrations compared to the non-sulfured PGC (Ma et al., 2017). Research indicates that sulfur fumigation modifies the metabolic profile of PGC in the intestines and blood of rats. Rats treated with non-sulfured PGC excreted 18 sulfated derivatives of ginsenosides and 26 related metabolites in their feces, along with six sulfated derivatives found in their plasma. In contrast, these components were undetectable in rats treated with sulfured PGC (Zhu et al., 2015). Moreover, sulfured PGC and PQL (Xi Yang Shen) may induce hepatorenal toxicity. Stored sulfured PGC can cause a decline in liver function indicators (ALT, AST), a decrease in CREA, and liver tissue degeneration in rats, showing stronger toxicity than non-sulfured PGC (Zhou et al., 2019). Sulfur-fumigated PQL generates SCDs that interact with metabolic pathways and key kidney targets, causing nephrotoxicity. Specifically, SCDs disrupt glyceride metabolism and the biosynthesis of phenylalanine, tyrosine, and tryptophan, leading to significant changes in metabolite levels related to kidney injury, such as increased fumarate and 2-heptanone. Furthermore, SCDs bind to key kidney targets (DECR1, PLA2G1B, and CAT), potentially causing mitochondrial dysfunction, oxidative stress, and cell damage, thereby inducing nephrotoxicity (He et al., 2023).

The immunomodulatory activity was evaluated using an ICR mouse model to compare the effects of non-sulfured PGC and sulfured PGC under normal conditions and in cyclophosphamide (CY)-induced immunosuppressed conditions. In normal mice, neither non-sulfured PGC nor sulfured PGC treatment showed significant immunomodulatory effects. In CY-induced immunosuppressed mice, non-sulfured PGC significantly alleviated the increase in spleen weight index, the elevation of serum IL-2 and IFN-γ levels, and the decrease in white blood cell count caused by CY, while sulfured PGC did not have these effects. Sulfur fumigation weakened the immunomodulatory activity of PGC, especially under immunosuppressed conditions (Ma et al., 2017).

ADBH (Bai Zhi) plays a crucial role in inflammatory diseases, with multiple components exhibiting anti-inflammatory properties (Zou et al., 2022). *In vitro* experiments show that both sulfured ADBH and non-sulfured ADBH have anti-inflammatory effects, but non-sulfured ADBH is more effective in reducing pro-inflammatory cytokines (e.g., TNF-α, IL-6, IL-1β). However, high-concentration sulfured ADBH still retains some anti-inflammatory activity (Wang C. et al., 2024).

Overall, current research on sulfur fumigation impacts focuses on only a few botanical drugs, lacking systematic comparisons across different botanical drug types. Most studies also fail to adequately account for the effects of botanical drug origin and variety differences on sulfur fumigation responses. Future research requires more rigorous experimental designs, broader scope, and the integration of multi - omics technologies to deeply explore sulfur fumigation mechanisms.

## 3 Traditional detection methods

After sulfur fumigation of TCM, the residual substances are typically quantified as SO_2_ residues. Since SO_2_ readily hydrolyzes in biological environments, it primarily exists in the form of sulfites (SO_3_^2-^) and bisulfites (HSO_3_^−^) within the medicinal materials (Peng et al., 2022). Since the release of the 2010 edition of the Chinese Pharmacopoeia, the process for determining SO_2_ residues in TCM has been standardized. This edition was notable for introducing a method for measuring SO_2_ residues through redox titration in its appendix (NLM, 2010). The subsequent 2015 edition further enhanced and refined the determination methods, incorporating three techniques: acid–base titration, ion chromatography, and gas chromatography (NLM, 2015). These methodologies have continued to be utilized in the 2020 edition of the Chinese Pharmacopoeia (NLM, 2020). Additionally, other methods for measuring SO_2_ residues include colorimetry (Bener et al., 2020), chromatography (Zhong et al., 2012), electrochemical methods (Zeng et al., 2015), and enzyme-linked photometric analysis (Lu et al., 2015). Current research on detection - method optimization mostly focuses on improving sensitivity, but neglects the methods’ selectivity and specificity. In complex TCM, accurately distinguishing sulfur - fumigation residues from other endogenous sulfur - containing substances remains a big challenge. Table 2 summarizes the SO_2_ detection methods, application scope and limits in different national standards. As shown in the table, different countries exhibit slight differences in the determination methods of sulfur dioxide residues, and their application scope also varies somewhat from that of China. Additionally, the provisions for limit standards are another key aspect reflected in the table.

**TABLE 2:** SO_2_ detection methods, application scope and limits in different national standards.

Country	Detection method of SO_2_	Application scope	Residue requirements for SO_2_
China	Titration method, ion chromatography method, gas chromatography methodetc.	Traditional Chinese Medicinal Materials and Prepared Slices	The Chinese Pharmacopoeia stipulates that the residual SO2 content in most traditional Chinese medicinal materials and preparations should be between 150–400 mg/kg, while no sulfur - fumigated products shall have no detectable SO_2_ residues
United States	Titration method, chromatography methodsetc.	Food	The FDA stipulates that foods with sulfite usage exceeding 10 mg/kg must be labeled accordingly
Europe	Ion chromatography method, gas chromatography methodetc.	Food, herbs and spices	Under EU Directive No 337/79, the residual SO2 in wine should be 150–200 mg/L. The residual SO2 in herbs and spices should not exceed 150 mg/kg. And the residual SO2 in dried vegetables like mushrooms, beans, algae, and in seed products should not exceed 500 mg/kg
Korea	Monier-Williams modification method	Herbs and spices	In the Korean standard for the use of food additives, the amount of sulfite residues in herbs and spices should not exceed 150 mg/kg in terms of SO2

Moreover, many conventional analytical methods face significant limitations. In under-resourced regions, implementing standards for determining SO_2_ residues faces two major bottlenecks. First, financial constraints make it difficult to purchase and maintain costly detection instruments like ion chromatographs and gas chromatographs. Second, there is a shortage of technical expertise, as trained personnel are needed to ensure the accuracy and reliability of test results. These factors hinder their effectiveness in rapid analysis (Mi et al., 2023). Also, these methods primarily focus on quantifying the residual SO_2_ and cannot directly reflect the duration of fumigation or the storage time. Research has shown that the SO_2_ residue in medicinal materials increases with prolonged fumigation time. However, after a certain period of storage, the residue tends to decrease. While chemical metabolites of the medicinal materials are altered during the fumigation process, these changes are not fully reversible, especially after the SO_2_ residue decreases during storage (Duan et al., 2016; Zhou et al., 2019). Therefore, measuring SO_2_ residues alone cannot comprehensively reflect the quality changes in medicinal materials after sulfur fumigation, nor can it adequately assess the associated risks.

## 4 Advances in novel detection technologies

### 4.1 Rapid detection techniques

Rapid detection technologies for SO_2_ residues in TCM have become a focal point for researchers dedicated to ensuring the quality and safety of medicinal materials. One notable technology that has gained significant attention is the use of lead acetate test strips. This method allows for the accurate quantification of SO_2_ residues in medicinal materials within minutes, effectively minimizing interference from the natural color of the materials. It provides a stable, reliable, convenient, and rapid detection solution. The underlying chemical principle involves the reduction of SO_2_ to hydrogen sulfide using sodium borohydride, which is subsequently detected with lead acetate test strips. The experimental procedure entails weighing 0.4 g of the medicinal material sample, adding it to a reaction vessel containing 2 mL of deionized water, adjusting the pH, mixing thoroughly, inserting a lead acetate test strip, adding sodium borohydride, sealing the vessel, and finally adding hydrochloric acid to facilitate the reaction. A detector equipped with a light source, imaging, and image analysis systems is then employed to analyze the test strip and obtain results (Zhao et al., 2024).

Fluorescent probe-based rapid detection technologies have also been extensively explored for the detection of SO_2_ and its derivatives. Fluorescent probes are preferred due to their ease of use, rapid response, high sensitivity, and excellent selectivity (Yang et al., 2019), leading to the development of numerous fluorescence-based sensing methods (P et al., 2023). However, the chemical similarity between thiols and SO_2_ derivatives presents a challenge for the selective detection of sulfur dioxide. To overcome this, a coumarin-derived fluorescent probe sensor for SO_2_ has been developed. By adjusting the pH of the reaction system, this probe can selectively identify HSO_3_^−^ based on the differing pKa values between HSO_3_^−^ and thiols, thus achieving selective detection of HSO_3_^−^ without interference from thiols (Mi et al., 2023). Researchers have also integrated this probe into a disposable test tube that combines test strips and centrifuge tubes, facilitating rapid visual and semi-quantitative detection of SO_2_.

Additionally, a fluorescent probe, PT1, activated under acidic pH conditions, has been designed for detecting SO_2_ and its derivatives in traditional Chinese medicines and lysosomes. PT1 offers rapid detection with ease of operation, low cytotoxicity, high photostability, reversible pH switching, effective lysosomal targeting, and narrow near-infrared emission under acidic activation (Xi et al., 2024).

Notably, fluorescent probes, despite their advantages of speed and sensitivity, still require selectivity enhancement. The currently developed probes are susceptible to interference from other fluorescent substances in medicinal materials and exhibit poor stability in complex biological matrices, which may affect the reliability of detection results.

### 4.2 Targeted marker analysis

Monitoring only SO_2_ residues is limited as it fails to reflect the quality, efficacy, and toxicity changes of sulfur-fumigated TCM. Using SO_2_ residue levels alone is insufficient for quality control (Zhou et al., 2019). It is suggested to identify sulfur-containing markers, such as sulfate/sulfite derivatives of bioactive metabolites from the fumigation process. Detecting these markers’ relative levels can assess the impact on the chemical composition of TCM, determining its safety and efficacy. Table 3 lists the targeted markers of sulfur-fumigated TCM.

**TABLE 3:** Targeted markers of sulfur-fumigated TCM.

TCM	Targeted markers of sulfur-fumigated TCM	References
*Trichosanthes kirilowii* Maxim. (Gua Lou Gen)	Cucurbitacin D sulfite I, Cucurbitacin D sulfite II, Cucbituracin B sulfite I, and Cucurbitacin B sulfite II	Kang et al. (2020)
*Atractylodes macrocephala* Kitag. (Bai Zhu)	Atractylenolide III sulfate、Isoasterolide A sulfite、6-hydroxy atractylenolideⅠsulfate et.	Sun et al. (2017)
*Moutan Cortex* Andr (Mu Dan Pi)	Paeoniflorin sulfate, quercetin and its glycoside derivatives, gallic acid and its derivatives	Zhan et al. (2018)
*Gastrodia elata* Bl. (Tian Ma)	p-hydroxybenzyl hydrogen sulfite and p-mercaptobenzyl hydrogen et.	Kang et al. (2017)
*Tussilago farfara* L. (Kuan Dong Hua)	Sulfur-containing phenolic derivatives	Wang et al. (2021)
*Angelica dahurica* (Fisch. ex Hoffmanns. and Link) Benth. and Hook.f. (Bai Zhi)	Ligustilide and oxypeucedanin	Wang C. et al. (2024)
*Achyranthes bidentata* Blume (Niu Xi)	Betavulgarosides II-IV,Feruloyl-4-O-methyldopamine and Moupinamide	Dong et al. (2023)
*Zingiber officinale* Rosc. (Sheng Jiang)	6-Ginger sulfonic acid	Wu et al. (2018)
*Lonicera japonica* Thunb. (Jin Yin Hua)	chlorogenic acid sulfate、secologanic acid sulfate、Chlorogenic acid sulfite、Secologanic acid sulfite	Shengyun et al. (2019)
*Paeonia lactiflora* Pall. (Bai Shao)	Paeoniflorin sulfate and paeoniflorin sulfite	Kong et al. (2014)

Many studies have explored targeted markers to identify sulfur-fumigated TCM. For instance, particular markers were identified after sulfur fumigation in AMK (Bai Zhu). They developed a UPLC-MS method to detect sulfur-fumigated AMR in the TCM formula Liu-Wei-Di-Huang-Wan, enabling quick identification of its presence in the formula (Li et al., 2017). Another study established a UHPLC-Q-Orbitrap HRMS method to identify sulfur-fumigated TFL (Kuan Dong Hua). Researchers identified diagnostic ions, including sulfated product ions and neutral losses, which facilitate the rapid screening of sulfur-fumigated samples (Wang et al., 2021).

For GEB (Tian Ma), p-hydroxybenzyl hydrogen sulfite (p-HS) was identified as a specific marker after sulfur fumigation (Kang et al., 2017). Its detection can indicate whether GEB has been sulfur-fumigated. A study employed capture-SELEX to select aptamers with high affinity for p-HS. After eight rounds, an aptamer (seq 6) with a Kd of 26.5 μM was obtained and characterized by ITC. Using this aptamer as a recognition element and gold nanoparticles (AuNPs) as a colorimetric indicator, a simple and efficient colorimetric sensor was developed for p-HS detection. With a detection limit of 1 μg/mL and a recovery rate of 88.5%–105% in market-purchased GEB samples, the sensor, based on a nucleic acid aptamer and a colloidal gold detector, enables rapid identification of sulfur-fumigated GEB (Wan et al., 2024). These studies validate the feasibility of specific marker detection in assessing sulfur-fumigated botanical drugs and provide scientific groundwork for more comprehensive detection methods.

### 4.3 Machine learning applications

Machine learning has been widely used to distinguish between sulfur - fumigated and non - sulfur - fumigated samples. Researchers first collect a certain number of such samples, then use spectroscopy or imaging technology for data collection and chemometrics for calculations. After that, various machine learning methods like K - nearest neighbors (KNN), logistic regression (LR), and back - propagation artificial neural networks (BP - ANN) are applied to build models, which are assessed using validation samples and finally used for qualitative or quantitative detection of the samples.

Near - infrared spectroscopy (NIR) has been used in this field. For example, in the detection of *Citrus reticulata* Blanco [Rutaceae; *Citri reticulatae* exocarpium rubrum] (CER) (Ju Hong), a portable NIR combined with chemometrics has established an accurate, non - destructive method for detecting sulfur - fumigated CER (SCER). With 389 normal CER and 350 SCER samples, the spectra of the exocarp and endocarp can be directly obtained without damaging the samples. New variable selection - linear discriminant analysis (LDA) and other LDA - based pattern recognition methods, along with spectral preprocessing, are used to build identification models. These models are evaluated using validation sets and external validation sets collected a month later. Results show that the exocarp has a higher recognition accuracy than the endocarp. The LDA model combining second - order derivatives with competitive adaptive reweighted sampling (CARS) performs best, with a validation set accuracy of 98.92% and an external validation set accuracy of 99.46% (Qiu et al., 2024). Similarly, NIR technology combined with chemometrics has successfully enabled rapid, non-destructive detection and visualization of SO_2_ residues in sulfur–fumigated *Fritillaria thunbergii* Siebold et Zucc. [Liliaceae; *Fritillariae thunbergii* bulbus] (Zhe Bei Mu). The study uses partial least squares regression (PLSR) models and various optimal wavelength selection methods, such as the successive projections algorithm and weighted regression coefficient method, to reduce data volume while maintaining model performance (He et al., 2017). A study utilizing NIR and chemometrics has effectively developed qualitative and quantitative analysis methods that do not require special sample extraction or chemical reagents. These methods can reliably distinguish between non-sulfur-fumigated and sulfur-fumigated ZOR (Sheng Jiang) (Yan et al., 2021).

In addition, image - processing - based methods have also achieved good results. A study has developed an efficient and convenient method to identify sulfur-fumigated ZOR (Sheng Jiang). First, a rapid test kit is used to mark three levels of sulfur - fumigated samples, and images of each level are collected. Then, brightness and texture features are extracted from the images, and three machine learning methods - support vector machines, BP - ANN, and random forests - are applied to build prediction models. The accuracy of each model is calculated, and different weights are assigned to them. Finally, a weighted voting mechanism determines whether the samples have been sulfur - fumigated, establishing the final identification model, which has a prediction accuracy of between 80% and 100% (Wang T. et al., 2023).

Spectroscopy and chemometrics combined with machine learning are widely used, too. In a study on AMB (Huang Qi), UV - vis - SWNIR DRS and chemometrics were used to determine SO_2_. Models showed high prediction accuracy (Jing et al., 2017). For distinguishing different sulfur - fumigated LJT (Jin Yin Hua) samples, a hyperspectral imaging - and - chemometrics - based technology was developed. After PCA of spectral data, classification models were built. The Least Squares Support Vector Machine model with optimal wavelengths selected by the CARS algorithm had the best performance, with an R^2^P of 0.9109 and an Root Mean Square Error of 0.3353 (Liu et al., 2018). In PGC (Ren Shen) detection, Fourier-Transform Infrared (FT–IR) spectroscopy and multivariate statistical analysis were used. Original spectra of 240 batches of sun - dried and sulfur - fumigated PGC were collected. Analysis showed FT - IR spectroscopy in the 3,650–3,200 cm^-1^ range can detect sulfur - fumigated PGC. BP - ANN had the highest model parameters, with accuracy at 91.67%, precision at 89.29% (Li et al., 2022).

It is worth noting that the construction of machine learning models is highly dependent on the quality and representativeness of the input data. However, the data used to train these models often come from small - scale laboratory experiments, which are less diverse than the medicinal materials in actual production and distribution. This may result in poor generalization of the models in practical applications. Also, some studies lack a solid basis for choosing machine learning algorithms and fail to thoroughly compare their suitability and pros/cons.

## 5 Alternative processing methods

### 5.1 Development of safe drying techniques

The use of sulfur fumigation in TCM is a widely recognized processing technique. This method effectively prevents mold and insect damage while enhancing the visual appeal of the materials. However, it poses certain hazards, as it alters the chemical composition of the materials, which can compromise their efficacy and safety. Consequently, the pursuit of safe and efficient alternative methods has emerged as a vital focus in contemporary research. Currently, various drying and sterilization techniques have been developed and implemented to substitute sulfur fumigation. These include natural drying, hot-air drying, vacuum drying, microwave drying, ultrasound-assisted drying, pulsed electric field pretreatment, and osmotic dehydration. Such techniques can significantly lower the moisture content of medicinal materials, reduce microbial contamination, and preserve their active components and pharmacological properties to varying extents (Wang D. et al., 2023). Natural drying and hot-air drying are widely utilized methods due to their cost-effectiveness and operational simplicity. However, these techniques can be time-consuming and inefficient, potentially resulting in the loss of active components during the drying process. In contrast, vacuum drying and microwave drying technologies provide enhanced drying efficiency and superior retention of product quality. Vacuum drying accelerates water evaporation by lowering pressure, making it suitable for heat-sensitive medicinal materials. Microwave drying, on the other hand, rapidly eliminates moisture through the thermal and dielectric effects of microwaves, while also offering a degree of sterilization. Additionally, emerging technologies such as ultrasound-assisted drying and pulsed electric field pretreatment further enhance drying efficiency and product quality by improving mass and heat transfer (Jin et al., 2017). Moreover, hybrid drying technologies, such as solar-assisted and microwave-assisted drying, demonstrate significant potential in various applications. These methods leverage the benefits of multiple drying techniques, enhancing efficiency and minimizing energy consumption while preserving the inherent qualities of medicinal materials. For instance, solar-assisted drying harnesses renewable energy sources, making it both environmentally friendly and sustainable. On the other hand, microwave-assisted drying accelerates the drying process and mitigates nutrient loss through rapid and uniform heating (Yang et al., 2024).

Some studies have explored how different drying technologies affect themulticomponent substances in TCM during drying. But current research still has many limitations. It’s found that hot air drying and medium-and-short-wave infrared drying are not only efficient but also can significantly increase the content of total flavonoids, total volatile oil, aldehydes, and alkenes in dried CER(Ju Hong) (Sun et al., 2023). What’s more, hot air drying consumes less energy. Hot air drying at 60°C is remarkable for drying CER (Ju Hong) and *Saposhnikovia divaricata* (Turcz.) Schischk. [Apiaceae; *Saposhnikoviae divaricatae* radix] (SDS) (Fang Feng), it can effectively preserve the active components of medical materials. At this time, the content of index components and the comprehensive evaluation score of SDS are the highest (Sun et al., 2024). When drying *Curcuma longa* L. [Zingiberaceae; *Curcumae longae* rhizoma] (Jiang Huang), a light-free condition at 70°C is better. It can retain the curcuminoids, color, total phenolic content, and antioxidant capacity of turmeric to the greatest extent (Komonsing et al., 2022). In the study of *Amomum tsao-ko* Crevost et Lec. [Zingiberaceae; *Amomi tsao-ko* fructus] (Cao Guo), it was found that electric baking at 50°C is conducive to obtaining a higher content of volatile oil (Qin et al., 2021). However, boiling water treatment and microwave heating and drying would reduce the volatile oil content and cause fruit cracking. As for *Crocus sativus* L. [Iridaceae; *Croci sativi* stigma] (Zang Hong Hua), freeze-drying is the most effective in maintaining its quality (Bashir et al., 2025). It can significantly increase the content of crocin, safranal, and crocetin. In addition, it performs better in maintaining color, anthocyanin content, antioxidant capacity, and microstructure integrity. However, most current research focuses on a few kinds of traditional Chinese medicine. There is a lack of a comprehensive and all-encompassing evaluation system. Most studies only pay attention to the changes in individual aspects, such as the content of specific active components or volatile compounds. It is difficult to comprehensively consider multi-dimensional factors such as the appearance quality, internal components, pharmacological activity, and safety of medicinal materials. As a result, the overall evaluation of drying technologies is not comprehensive enough. In addition, there are significant differences in the parameter settings of the same drying technology in different studies. Temperature, time, humidity, and other conditions are not unified, making it difficult to compare research results and draw conclusions with universal applicability. What’s more, most existing studies only focus on the changes of multicomponent substances in TCM in the short term after drying. There are few studies on the stability of components and the maintenance of biological activity during long-term storage. Thus, it is hard to accurately evaluate the impact of different drying technologies on the long-term quality of TCM.

### 5.2 Desulfurization technologies

Thermal desulfurization plays a vital role in eliminating residual sulfur from TCM that have undergone sulfur fumigation. This method offers significant advantages in reducing SO_2_ residues. For instance, in sulfur-fumigated CPF (Dang Shen), this technology effectively decreases SO_2_ levels, thereby mitigating health risks associated with excessive SO_2_ exposure (Xu et al., 2020). Despite its advantages, this process does come with certain drawbacks. While it successfully reduces SO_2_ residues, it also alters the chemical composition of the botanical drugs, which have already been modified by sulfur fumigation. Certain bioactive compounds, diminished by sulfur fumigation, experience further reductions with thermal desulfurization, and the polysaccharide content declines markedly. In the case of MCA (Mu Dan Pi) desulfurization, SO_2_ residues decrease from over 1,400 μg/g to below 120 μg/g, complying with the Chinese Pharmacopoeia standard (Zhan et al., 2018). Nevertheless, sulfur fumigation generates new sulfur-containing compounds in MCA, which cannot completely revert to their original glycosides during desulfurization. Additionally, this process eliminates some water-soluble compounds, including paeoniflorin sulfite, paeoniflorin, and methyl gallate. In conclusion, while thermal desulfurization is effective in reducing SO_2_ residues, it significantly impacts the chemical composition and potential efficacy of the botanical drugs. Striking a balance between ensuring botanical drug safety and preserving their natural components and efficacy remains a critical challenge in the processing of traditional Chinese medicine.

## 6 Future directions

### 6.1 Research priorities

#### 6.1.1 Further exploration of sulfur-induced chemical transformations

Sulfur fumigation has the potential to alter the chemical composition of TCM, which may compromise their efficacy and safety. Future research should explore the specific reactions that occur during sulfur fumigation and their impact on the quality and safety of these materials. In sulfur fumigation -related chemical changes, focus on the dynamic changes of medicinal material metabolites during fumigation. Use real-time monitoring technology and combine pharmacokinetic and pharmacodynamic research to clarify the time-dependent effects of sulfur fumigation on the *in-vivo* processes and pharmacological effects of medicinal materials. Also, strengthen cross-disciplinary collaboration, introduce computational chemistry methods to predict the biological activity and toxicity of sulfur - fumigated products, and offer theoretical support for risk assessment.

#### 6.1.2 Validation of novel detection technologies and alternative methods

As technology progresses, innovative detection technologies and alternative processing methods are continually emerging. It is crucial to systematically validate and assess these advancements to ensure their effectiveness and reliability in practical applications. This process involves testing the performance of new drying and sterilization technologies, as well as thoroughly evaluating the quality of medicinal materials to confirm compliance with industry standards and regulatory requirements. For instance, hyperspectral imaging can facilitate the rapid detection of sulfur dioxide residues in medicinal materials, while Raman spectroscopy can provide real-time monitoring of chemical changes during the drying process. Furthermore, novel drying technologies, such as pulsed electric field-assisted drying and supercritical fluid drying, must undergo extensive experimental validation to ascertain their feasibility and advantages in the drying of medicinal materials. In the detection technology and alternative method validation, large-scale, multi - center collaborative studies are urgently needed to rigorously validate novel detection technologies and alternative processing methods. For alternative processing method, future research should cover more TCM and study the changes in their multi - component substances under different drying technologies. It's essential to build a comprehensive evaluation system covering the appearance, chemical composition, pharmacological activity, and safety of medicinal materials, enabling a thorough and objective assessment of drying technologies. Based on this, unified experimental schemes and operational standards should be established. Key parameter settings for various drying technologies must be clarified to minimize experimental condition - induced deviations, enhancing research comparability and reliability and facilitating communication and integration among studies. Long - term storage experiments on dried TCM should be strengthened. Monitoring the changing trends of multi - component substances during storage can evaluate the role of different drying technologies in ensuring the long - term stability of TCM. Finally, in - depth exploration into the internal mechanisms behind the changes in multi - component substances of TCM during the drying process is needed. Explaining the principles of different drying technologies at the molecular level can provide a solid foundation for developing new drying technologies and processes.

### 6.2 Policy and regulation-establishment of stricter standards for sulfur fumigation and residue limits

The current regulatory standards governing sulfur-fumigated medicinal materials are relatively lenient, resulting in a significant presence of over-fumigated products in the market. To ensure the safety and quality of these medicinal materials, it is essential to implement stricter standards for sulfur fumigation and residue limits. Achieving this goal necessitates collaborative efforts among governments, industry organizations, and research institutions to establish scientifically sound and reasonable standards, as well as to enhance regulatory enforcement for strict compliance. For instance, by referencing international standards and leveraging the experiences of advanced countries, we can formulate sulfur dioxide residue limits that are appropriate for China’s unique national conditions. Additionally, improving market monitoring and regulation, coupled with imposing strict penalties for violations, will elevate the industry’s awareness regarding quality and compliance.

## 7 Conclusion

This study highlights that sulfur fumigation, while playing a positive role in preventing mold and insect pests in traditional Chinese medicine (TCM), may trigger chemical reactions that reduce efficacy and pose safety risks. These changes are mainly manifested in the alteration of the concentration and structure of bioactive compounds, as well as the formation of toxic derivatives. Such alterations not only weaken the therapeutic effects of botanical drugs but also pose potential threats to human health. Therefore, under the modern medical emphasis on safety and efficacy, it is necessary to reevaluate the necessity of the traditional sulfur fumigation process.

Emerging drying technologies such as vacuum drying, microwave drying, and ultrasonic-assisted drying have become viable alternatives to sulfur fumigation. These technologies offer significant advantages in ensuring the quality and safety of medical materials. However, their widespread application faces challenges such as high equipment costs, high demands for specialized skills, and difficulties in scaling up.

Developing sulfur fumigation residue detection technologies that comply with regulatory standards is of great importance. This involves not only the detection of SO_2_ residues but also the identification of specific sulfur markers to assess the degree of chemical changes in botanical drugs. Rapid detection technologies combined with machine learning and chemometrics show great potential in enhancing the accuracy and reliability of detection.

Given the significance of TCM to public health, the establishment of stricter regulatory standards for sulfur fumigation and its residues is urgent. The current standards are relatively lenient, allowing over-fumigated products to enter the market. By referring to international standards and drawing on the experience of advanced countries, it is possible to formulate more practical residue limits that fit China’s actual conditions. Strengthening market monitoring and regulation, and increasing penalties for violations, will effectively enhance the industry’s focus on quality and compliance.

In summary, addressing the quality issues of sulfur-fumigated medicinal materials is of utmost urgency. The coordinated advancement of innovative drying technologies, advanced detection methods, and strict regulatory frameworks points the way for industry development. Future research should not only focus on technological innovation but also pay attention to its socio-economic impacts, ensuring that the transition to safer practices is both efficient and equitable. The ultimate goal is to enhance the international competitiveness of TCM, promote the modernization and sustainable development of TCM industry, and contribute to public health and wellbeing.
